# A Rosetta Stone for Brain Waves

**DOI:** 10.1371/journal.pbio.1001063

**Published:** 2011-05-17

**Authors:** Mason Inman

**Affiliations:** Freelance Science Writer, Berkeley, California, United States of America

**Figure:**
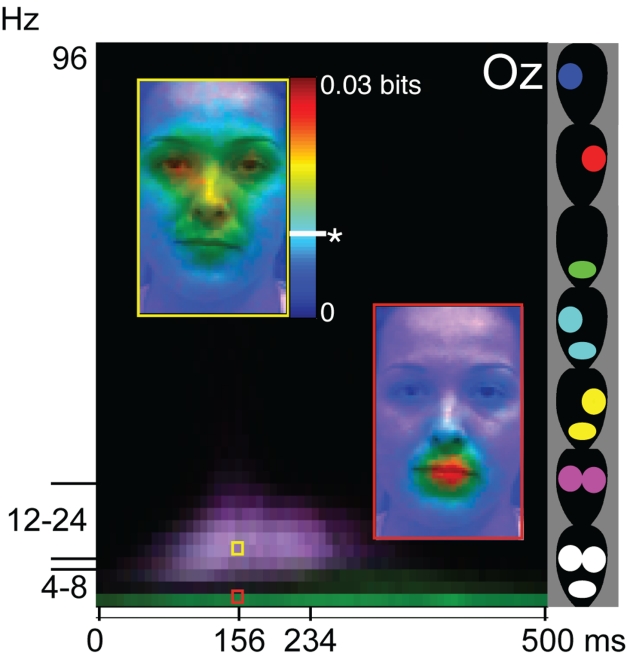
The colored boxes illustrate that different brain waves (4–8 Hz versus 12–24 Hz) act as coding channels for different facial information, increasing coding capacity.

Whenever we watch the world around us or dream about it when sleeping, our neurons produce “brain waves”—coordinated patterns of firing that create electrical signals that repeat in regular patterns.

But exactly what messages are encoded in these brain waves, and how they are encoded, is still largely unresolved. The problem is that the brain has its own language for sending and receiving signals. Researchers have made great strides in decoding many parts of this language, but some are still largely mysterious—including the messages in brain waves.

The Rosetta Stone allowed archeologists to decode Egyptian hieroglyphics by showing how those characters matched up with letters in the well-known script of ancient Greek. Similarly, a new study by Philippe Schyns and colleagues at the Institute of Neuroscience and Psychology at the University of Glasgow takes some initial steps toward cracking the brain wave code by matching up these waves with well-studied behavior: how people respond to looking at faces.

The team recruited six volunteers and presented each of them with images of people's faces displaying basic emotions such as happiness, fear, and surprise. The images were partially covered with randomly generated masks, so that the volunteers might see only the eyes and part of the mouth, for example, and were asked to say what emotion they saw in the image. The team then recorded whether they were correct or not. While doing these face-recognition tests, each volunteer's brain waves were being measured by electroencephalography (EEG), using a cap with several dozen electrodes touching their scalp.

By coordinating the brain wave signals with the photos of faces that the subjects saw, and the volunteers' responses about what emotion they thought they saw in the photos, then Schyns and colleagues were able to build a sort of Rosetta Stone for brain waves.

The brain has different frequencies of common waves—such as “theta” waves around 4 hertz (Hz), which repeat every one-fourth of a second, and “beta” waves at 12 Hz. The researchers found that the brain oscillations at certain frequencies tended to carry certain information about the face—just as one TV channel might carry mostly sports shows, and another channel mostly news. In one case, for example, beta waves encoded two eyes and theta waves encoded the mouth. By using multiple frequencies to encode two different parts of the face—a process known as multiplexing—then the brain can send more signals at the same time, just as having multiple TV channels allows the airwaves to carry more information at a time.

Schyns and colleagues also found that within each kind of wave, the information could be encoded in more than one way. One type of encoding is in the timing, or “phase,” of the wave. If delayed somewhat from the brain's baseline hum at that frequency, it relays some information, with a delay represented by degrees between 0° and 360° (like how a clock's minute hand sweeps through 360° in the course of an hour). Beta waves encode eyes using a phase delay of between 45° and 90°, the study found, whereas theta waves encode the mouth with a phase delay between 270° and 315°.

The study also found that the brain can encode information in the amplitude of the oscillations as well. Using statistical measures to estimate the strength of connection between volunteers' responses and accompanying brain waves, the researchers determined which aspects of the brain waves encoded the most information. Variations in phase encoded 2.4 times more information than variations in amplitude, they found. And when brain waves combined both phase and amplitude, they encoded three times more information than they did in amplitude alone.

By showing the key role of phase in encoding information, and by teasing apart the contributions of the various ways that the brain encodes information—including amplitudes, phases, and frequencies—Schyns and colleagues hope to open a new path to deciphering the brain's oscillations.

**Schyns PG, Thut G, Gross J (2011) Cracking the Code of Oscillatory Brain Activity. doi:10.1371/journal.pbio.1001064**

